# Effect of *Panax ginseng* on preventing acute respiratory tract infection: A protocol for systematic review and meta-analysis

**DOI:** 10.1097/MD.0000000000020690

**Published:** 2020-06-12

**Authors:** Zepeng Zhang, Peng Xu, Zhihong Wang, Daqing Zhao, Qingxia Huang, Jing Lu, Liwei Sun, Jian Wang, Xiangyan Li

**Affiliations:** a Research Center of Traditional Chinese Medicine, College of Traditional Chinese Medicine, Changchun University of Chinese Medicine, Changchun; b College of Acupuncture and Tuina, Changchun University of Chinese Medicine; c Neurology Department, College of Traditional Chinese Medicine, Changchun University of Chinese Medicine, Changchun; d Institute of Acupuncture and Tuina, Changchun University of Chinese Medicine; e Jilin Ginseng Academy, Changchun University of Chinese Medicine, Changchun; f Jilin Provincial Key Laboratory of Bio-Macromolecules of Chinese Medicine, Changchun University of Chinese Medicine, Changchun, China.

**Keywords:** acute respiratory tract infection, *panax ginseng*, protocol, systematic review and meta-analysis

## Abstract

**Background:**

Acute respiratory tract infection (ARTI) should be deeply concerned all over the world. *Panax ginseng* (ginseng) as traditional Chinese medicine is widely used in the treatment and health care for respiratory diseases. However, only one similar systematic review based on common cold has been published in 2011. New studies have occurred and a new systematic evaluation which could describe ARTI is needed.

**Methods and analysis:**

We will search for randomized control trials of ginseng on preventing acute respiratory tract infection in the following 8 databases: Cochrane Central Register of Controlled Trials (CENTRAL), MEDLINE, EMBASE, AMED (via OVID) and 4 Chinese databases (Chinese Biomedical Literature Database, China National Knowledge Infrastructure, Chinese Science and Technology Periodical Database, and Wan fang Database). The time is limited from the construction of the library to April 2020. The selection of studies, data extraction and quality of assessment will be conducted independently by 2 reviewers. The morbidity of ARTI by assessing self-report, caregiver report or clinical confirmation will be considered as the primary outcome. ARTI-related death among children or adults, other adverse events, absenteeism, laboratory-confirmed infection will be regarded as secondary outcome. All reported side effects and adverse events will be included as safety outcomes. Standard meta-analysis will be performed using Rev Man software V5.3.

**Results:**

This study will provide a better understanding of the association between *P ginseng* and ARTI.

**Conclusion:**

This systematic review may offer stronger evidences for the clinicians to prevent the patients from ARTI and update the former one based on basic diseases and the safety.

**PROSPERO registration number:**

CRD42020181317.

## 1 Introduction

Current stage, the whole world suffered from a health crisis, Corona Virus Disease 2019 (COVID-19), an acute respiratory tract infection (ARTI) with high mortality rate.^[1]^ Influenza and other respiratory viral infections are the most common type of ARTI.^[2]^ According to statistics, the number of deaths caused by ARTI in China is nearly 100,000 every year.^[3]^ Meanwhile, ARTI also could increase the risk of acute attacks in patients with chronic obstructive pulmonary disease (COPD), cardiovascular disease and other chronic diseases.^[4–6]^

Medical technicians around the world have carried out many researches on different viruses and pathogens, but they still cannot effectively control the global pandemic such as COVID-19.^[7]^ In the theory of traditional Chinese medicine, the Qi can protect the human body from pathogenic factors like virus is called Defensive Qi (Wei Qi in Chinese).^[8]^ Especially patients suffering from chronic diseases will show a more significant trend of Qi Deficiency.^[9,10]^ Therefore, benefiting Qi is helpful to strengthen the immunity and resist the invasion of exogenous evils to human health.^[11,12]^

Ginseng is a plant in the family Araliaceae and the genus *Panax* with the formal name of *Panax ginseng C. A. Meyer* and the treasure of traditional herbal medicine resources as the “King of Herbs.”^[13,14]^ Ginseng is famous for its remarkable effect of benefiting Qi.^[15]^ The application of benefiting Qi herb such as *P ginseng* (ginseng) is the cornerstone of Traditional Chinese Medicine (TCM) in the prevention of exogenous diseases, such as ARTI.^[16]^ A series of clinical studies have found that ginseng has a preventive effect on ARTI, however, none of these studies could provide definitive evidence due to limited research design or small sample.^[17–19]^ Therefore, a high-quality systematic review and meta-analysis to summarize current clinical evidence is urgently needed.

After preliminary search and database analysis, we have found that there has been no relevant systematic review and meta-analysis for many years. The most recent study was published in 2011 and only included 5 studies.^[17]^ Therefore, we hope to evaluate the preventive effect of *P ginseng* or *P ginseng* extract on ARTI to provide sufficient evidence for medical personnel.

## 2 Methods

### 2.1 Study registration

The protocol of this systematic review and meta-analysis has been registered in the International Prospective Register of Systematic Reviews (PROSPERO), and the registration number is CRD42020181317. This systematic review and meta-analysis will be reported in accordance with the guidelines of the Cochrane handbook for systematic reviews of interventions and the preferred reporting items for systematic reviews and meta-analyses (PRISMA) statement. Ethical approval is not required for this study.

### 2.2 Inclusion and exclusion criteria

#### 2.2.1 Types of studies

Parallel-group randomized controlled trials (RCT) will be included. No restriction will be put on the language, publication date or status of the study.

#### 2.2.2 Types of patients

The target population is adult or children irrespective of gender and ethnicity with symptoms of ARTI. A clinical diagnosis of ARTI was the main inclusion criteria. Diagnoses of upper or lower ARTI include acute common cold, influenza, rhino sinusitis, laryngitis, tonsillitis, pharyngitis, croup, acute otitis media, bronchitis, pneumonia, and acute exacerbations of COPD.

#### 2.2.3 Types of interventions

The experimental interventions include a ginseng alone and a combination of ginseng and another active treatment (pharmacological or nonpharmacological intervention).

No restrictions will be made on the types of control groups

#### 2.2.4 Types of outcome measures

The primary efficacy end point is the frequency rate of ARTI.

##### 2.2.4.1 Primary outcomes

The primary outcome will be the morbidity of ARTI by assessing self-report, caregiver report or clinical confirmation.

##### 2.2.4.2 Secondary outcomes

ARTI-related death among children or adults, other adverse events, absenteeism, laboratory-confirmed infection will be regarded as additional outcome. And all reported side effects and adverse events will be included as safety outcomes.

### 2.3 Search methods for the identification of studies

#### 2.3.1 Electronic searches

Two investigators search the following databases from construction of databases to April 2020: MEDLINE, EMBASE, Cochrane Central Register of Controlled Trials (CENTRAL), the Cumulative Index to Nursing and Allied Health Literature (CINAHL), Web of Science and three Chinese databases (China BioMedical Literature, China National Knowledge Infrastructure, and Wan Fang database).

We will conduct search strategy by using Medical Subject Headings/Emtree headings combined with free text words. The search strategy for PubMed is shown in Table 1. The equivalent search words will be used in the Chinese databases.

**Table 1:**
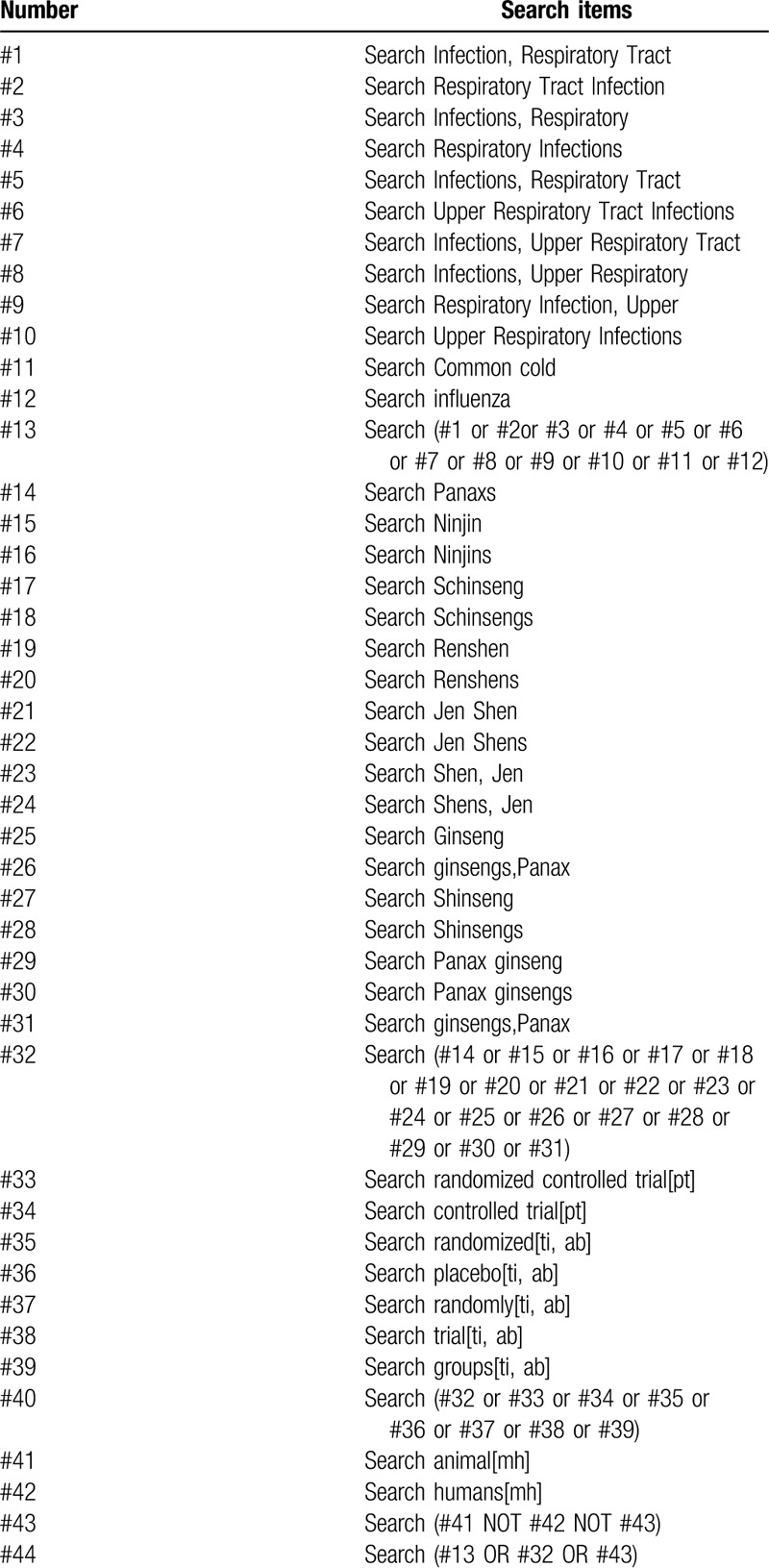
Search strategy used in PubMed database.

#### 2.3.2 Searching other resources

In addition, Chinese Clinical Trial Registry, Clinical Trials.gov, International Clinical Trials Registry Platform (WHO-ICTRP) will be searched to identify additional ongoing or unpublished studies.

### 2.4 Data collection and analysis

#### 2.4.1 Selection of studies

**Figure 1:**
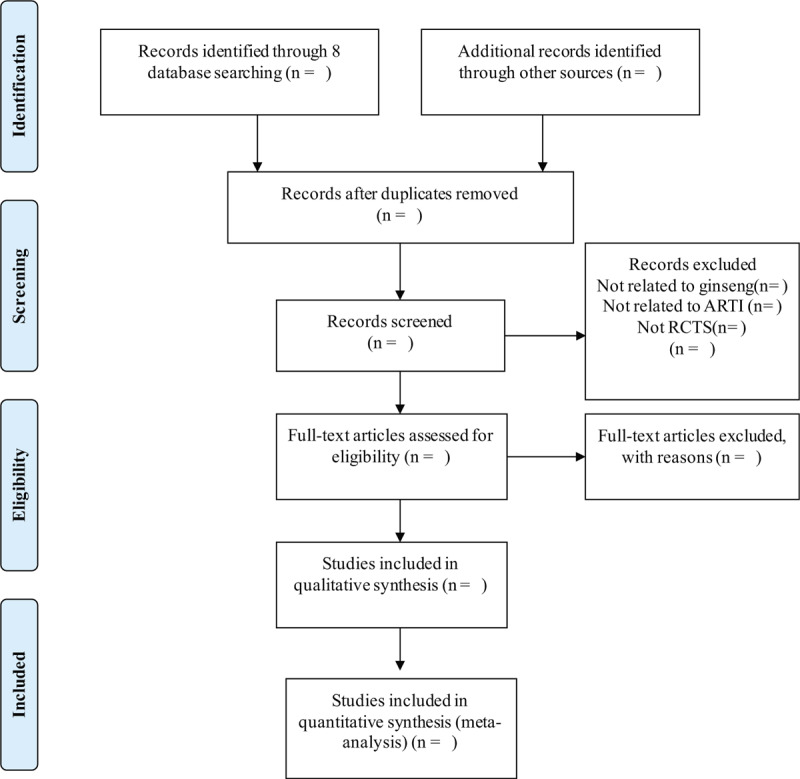
Flow chart of study selection. ARTI = acute respiratory tract infection, RCT = randomized controlled trials.

The titles and abstracts of studies retrieved using the search strategy and those from additional sources will be screened independently by 2 review authors to identify the studies that potentially meet the predetermined inclusion criteria. Disagreement in this and all following steps of the systematic review process will be resolved by discussion or adjudication by a third reviewer, when necessary. The authors will the record reasons for exclusion of each study and report the results of the screening, according to PRISMA flow diagram.^[20]^ The flow diagram of study selection was shown in Figure 1.

#### 2.4.2 Data extraction and management

A standard data extraction form will be created before data extraction. Two reviewers (JL and QH) will independently extract the following information:

1. General information (title, first author, year of publication, funding).
2. Study characteristics (design, randomization, allocation, blinding, inclusion and exclusion criteria, sample size).
3. Participant characteristics (age, ethnicity, diagnosis criteria, number in each group).
4. Intervention characteristics (intervention, comparator intervention, dos-age, frequency, and duration).
5. Outcomes (primary and secondary outcomes, time points, methods of outcome assessments, blinding of outcome assessment, adverse events).

Only the latest report will be included when a same trial was described by multiple publications. Data not available in the publications will be obtained by contacting corresponding authors for more information.

#### 2.4.3 Date synthesis

We will perform the meta-analysis when more than one trial examines the same intervention and outcomes with comparable methods in similar populations. If the statistical heterogeneity is not identified, the fixed-effect model will be built to estimate the overall intervention effects.^[21]^ Otherwise, the random-effect model will be used to provide more conservative results. When multiple intervention groups are used in a study, we will make pair-wise comparisons by combining groups if possible. All statistical analyses will be performed by the RevMan V.5.3 software. The statistical significance is defined as *P* < .05. If the meta-analysis is not feasible, we will provide a narrative description of the results.

#### 2.4.4 Risk of bias assessment

The methodological quality of each individual study will be independently assessed by 2 reviewers (ZW and DZ) according to the Cochrane ROB tool.^[22]^ The following seven domains will be assessed: random sequence generation, allocation concealment, blinding of participants and personnel, blinding of outcome assessment, incomplete outcome data, selective outcome reporting, and other potential sources of bias. The risk of bias for each domain will be graded as low, high or unclear for each included study. The consistency will be checked by a third reviewer (LS) and the disagreements were resolved by discussion with methodologists (JW, XL).

#### 2.4.5 Assessment of heterogeneity

Statistical heterogeneity across the studies included will be tested using *χ*^2^ test and *I*^2^ statistic. The heterogeneity is significant statistically when the *P* value based on *χ*^2^ test less than 0.10 or *I*^2^ more than 50%.^[23]^ If so, exploratory sensitivity or subgroup analyses will be performed to identify possible reasons.^[24]^

#### 2.4.6 Assessment of reporting biases

The reporting bias will be investigated using visual funnel plots if more than 10 RCTs are included in a meta-analysis. If the reporting bias is identified, we will explore possible reasons using the subgroup analysis or meta-regression analysis.^[25]^

#### 2.4.7 Assessment of evidence quality

The overall quality of the evidence will be assessed using the Grading of Recommendations Assessment, Development and Evaluation (GRADE) approach on the efficacy and safety of *P ginseng* for acute respiratory tract infection. The quality of RCT evidence will be classified into “high,” “‘moderate,” “low,” or “very low” quality evidence, depending on the presence of these 5 factors:

(1) limitations in the design and implementation;
(2) indirectness of evidence;
(3) unexplained heterogeneity or inconsistency of results;
(4) imprecision of results; and
(5) high probability of publication bias.^[25]^

### 2.5 Analysis of subgroups or subsets

Subgroup analyses will be divided by basic disease, literature quality and type of outcome reported.

## 3 Discussion

ARTI induced by COVID-19, influenza or chronic diseases causes the horrible threat to human health in the whole world. Based on the effect of benefiting Qi, ginseng can strengthen human immunity against ARTI, but the relationship between ginseng and the prevention of ARTI is still unclear. Some new studies have been conducted since 2011, it is necessary to update previous systemic review about the preventive effect of ginseng on ARTI. This study will obtain the evidences of ginseng or ginseng extract on ARTI prevention by summarizing previous clinical evidences.

The advantages of this review will be:

(1) this review will include more clinical studies than the former one, since the last study only extracted the proportion and symptoms of a cold or ARTI in only 5 research reports;
(2) this study will also try to analyze the research reports of ARTI from COPD, cardiovascular disease, and other chronic diseases and get more comprehensive and practical results;
(3) To avoid bias as much as possible, we will collect all relevant documents as comprehensively as possible. As to the exploration of heterogeneity, post hoc subgroup analysis should be avoided as much as possible.

Despite these efforts, the limitations in this systematic review will still exist:

(1) Only the studies for conventional treatment with ginseng will be included, the formula or others combined with ginseng will not be included;
(2) the origin and age of ginseng in included studies are not mentioned and should be considered for the analysis.

## Author contributions

**Conceptualization:** Zepeng Zhang, Peng Xu, Jian Wang, Xiangyan Li.

**Data curation:** Peng Xu.

**Formal analysis:** Qingxia Huang, Jing Lu.

**Funding acquisition:** Daqing Zhao.

**Investigation:** Zhihong Wang.

**Methodology:** Peng Xu, Liwei Sun, Xiangyan Li.

**Project administration:** Zepeng Zhang.

**Resources:** Zepeng Zhang, Peng Xu.

**Software:** Zepeng Zhang, Peng Xu.

**Supervision:** Jian Wang, Xiangyan Li.

**Writing – original draft:** Zepeng Zhang, Peng Xu.

**Writing – review & editing:** Jian Wang, Xiangyan Li.
